# Confidentiality breaches in clinical practice: what happens in hospitals?

**DOI:** 10.1186/s12910-016-0136-y

**Published:** 2016-09-02

**Authors:** Cristina M. Beltran-Aroca, Eloy Girela-Lopez, Eliseo Collazo-Chao, Manuel Montero-Pérez-Barquero, Maria C. Muñoz-Villanueva

**Affiliations:** 1 Section of Legal and Forensic Medicine, Faculty of Medicine and Nursing, University of Córdoba, Avenida Menéndez Pidal s/n, 14004 Córdoba, Spain; 2 Internal Medicine Department, IMIBIC/Hospital Reina Sofia, University of Cordoba, Córdoba, Spain; 3 Statistic and Methodology Department, IMIBIC, Córdoba, Spain

**Keywords:** Confidentiality/privacy, Professional ethics, Professional-patient relationship

## Abstract

**Background:**

Respect for confidentiality is important to safeguard the well-being of patients and ensure the confidence of society in the doctor-patient relationship. The aim of our study is to examine real situations in which there has been a breach of confidentiality, by means of direct observation in clinical practice.

**Methods:**

By means of direct observation, our study examines real situations in which there has been a breach of confidentiality in a tertiary hospital. To observe and collect data on these situations, we recruited students enrolled in the Medical Degree Program at the University of Cordoba. The observers recorded their entries on standardized templates during clinical internships in different departments: Internal Medicine; Gynecology and Obstetrics; Pediatrics; Emergency Medicine; General and Digestive Surgery; Maxillofacial Surgery; Plastic Surgery; Orthopedics and Traumatology; Digestive; Dermatology; Rheumatology; Mental Health; Nephrology; Pneumology; Neurology; and Ophthalmology.

**Results:**

Following 7138 days and 33157 h of observation, we found an estimated Frequency Index of one breach per 62.5 h. As regards the typology of the observed breaches, the most frequent (54,6 %) were related to the consultation and/or disclosure of clinical and/or personal data to medical personnel not involved in the patient’s clinical care, as well as people external to the hospital. As regards their severity, severe breaches were the most frequent, accounting for 46.7 % of all incidents. Most of the reported incidents were observed in public areas (37.9 %), such as corridors, elevators, the cafeteria, stairs, and locker rooms.

**Conclusions:**

In addition to aspects related to hospital organization or infrastructure, we have shown that all healthcare personnel are involved in confidentiality breaches, especially physicians. While most are committed unintentionally, a non-negligible number are severe, repeated breaches (9.5 %), thus suggesting a certain carelessness, perhaps through ignorance about certain behaviors that can jeopardize patient confidentiality.

**Electronic supplementary material:**

The online version of this article (doi:10.1186/s12910-016-0136-y) contains supplementary material, which is available to authorized users.

## Background

Medical professionals are obligated to protect the confidentiality of their patients. The duty to ensure discretion and confidentiality in the medical profession is morally justified based on the rights arising from relationships, and medical practice involves trust relationships with both patients and society. This duty of confidentiality provides a fundamental basis for the existence of some level of trust in the doctor-patient relationship [1, 2]. From the ethical point of view, respect for the principles of beneficence, non-maleficence and also autonomy is recognized as a major justification for maintaining patient confidentiality, based upon a fundamental consideration for persons [3]. Altisent [4] defines it as “the moral right to assist people in maintaining the privacy of what they entrust to others, who correlatively acquire the obligation to guard secrecy”.

Respect for confidentiality is important to safeguard the well-being of patients and ensure the confidence of society in the doctor-patient relationship. Health information is not only based on objective observations, diagnoses, and test results, but also subjective impressions about the patient, their lifestyle, habits, and recreational activities. The improper disclosure of such highly sensitive information could harm patients’ reputation or result in lost opportunities, financial commitments, and even personal humiliation [5]. This obligation is stringent but not unlimited. In fact, there are two general exceptions where it is necessary to question whether or not to maintain confidentiality: when the safety of others or public health is threatened [6, 7].

Medicine today is practiced by healthcare teams formed not only by physicians, residents, and nursing staff, but also nursing assistants, orderlies, administrative personnel, and even students. Patients should be aware of the large number of people in hospitals who need to access their medical records to provide the best possible health care [8], which consists in obtaining an accurate diagnosis, providing the appropriate treatment, as well as receiving the necessary training to do so. It is for this reason that hospital personnel are required to protect patient confidentiality. Breaches of confidentiality in clinical practice due to carelessness, indiscretion, or sometimes even maliciously, jeopardize a duty inherent in the doctor-patient relationship [9]. Careless behavior, such as speaking about patients in public spaces like elevators [10] and cafeterias, during telephone conversations, or even when accessing electronic data, can result in breaches of patient confidentiality [7].

By means of direct observation, our study examines real situations in which there has been a breach of confidentiality. To achieve our aim, we first estimate the frequency of the phenomenon, that is, we quantify the number of times that patient confidentiality is breached in the different medical departments of a hospital. We then classify the situations recorded by the observers according to two characteristics: type and severity. Thirdly, we establish a relationship between the data recorded during the observations: the specific medical department and area where the observations were made, and the type of professional involved. The identification and characterization of such situations could be of use to health professionals and hospital management with a view to implementing the necessary measures to prevent such incidents.

## Method

### Experimental design

We conducted an observational, cross-sectional epidemiological study on situations defined as breaches of confidentiality in clinical practice. The study was carried out in a 1197-bed university tertiary hospital with an average of 39,912 admissions and 748,245 patient visits per year.^1^

Research was conducted in compliance with the Helsinki Declaration and approved by the Ethics Committee of Clinical Research of the reference hospital.

Additionally, our study adheres to STROBE guidelines (Additional file 1) for reporting observational research. 2

### Selection of participants and sample collection

To observe and collect data on situations in which confidentiality was breached, we recruited 5^th^-year and 6^th^-year students enrolled in the Medical Degree Program at the University of Cordoba at the beginning of the academic years 2010–2011, 2011–2012, 2012–2013, and 2013–2014. All participants were adults, and signed a consent form with a confidentiality agreement, especially in order to avoid awareness of the study and consequently the bias of changing the behavior of the observed subjects. A total of 99 observers (75 women and 24 men) participated in the study, two of which abandoned the project.

To ensure the anonymity of the participants in the study, each of the observers was assigned a numerical code. In order to standardize the collection of data, the observers were trained by the researchers through interviews and in training sessions with groups of up to three students. A checklist was used during the training sessions to inform the observers about different types of confidentiality breaches. Specifically, the checklist contained several items describing situations in which the most common confidentiality breaches may occur. However, the observers were also instructed to record any other type of incident that was not specifically reflected on the checklist. Incidents that the researchers did not consider to be examples of unethical conduct (i.e., breaches of confidentiality) were excluded from the study.

The observers recorded their entries on standardized templates during clinical internships in the following departments and units: Internal Medicine; Gynecology and Obstetrics; Pediatrics and specialties; Adult Emergency Medicine; General and Digestive Surgery: Hepatobiliary Surgery, Colorectal Surgery, Breast Surgery, Endocrine and Upper Gastrointestinal Surgery, and Oncological Surgery; Maxillofacial Surgery; Plastic Surgery; Orthopedics and Traumatology; Digestive; Dermatology; Rheumatology; Mental Health; Nephrology; Pneumology; Neurology; and Ophthalmology.

In addition to describing each breach of confidentiality, the observers recorded the total number of days and hours corresponding to each period, the area/s where the breach occurred, the day and time of the incident, the type of health professional responsible for the breach, as well as the gender and age range of the person involved. It seems important to underline that observers were interested in collecting the type of professional, as well as another anonymous sociodemographic data; therefore, the identity of the observed subjects remained unknown for the researchers.

### Study variables

#### Medical departments

The medical departments in which the observations were made included a total of 37 Clinical Management Units (CMU). Due to the diversity of the units and the scarcity of data observed in some of them, we decided to regroup them into seven categories according to the similarities between them, especially when the rotation period of the students was less than 200 days. The resulting categories were:

1. Internal Medicine and the Emergency Department
2. Gynecology and Obstetrics
3. Pediatrics
4. General and Digestive Surgery
5. Maxillofacial Surgery and Plastic Surgery
6. The rest of the CMUs corresponding to other medical or surgical specialties were grouped into a single category that included the Orthopedics and Traumatology Department and the Emergency Department, as well as the Digestive, Dermatology, Rheumatology, Mental Health, Nephrology, Pneumology, Neurology, and Ophthalmology departments.
7. Finally, an additional “Unknown” category included breaches of confidentiality observed in other areas of the hospital or committed by personnel who did not belong specifically to any CMU or medical department.

### Number of observations

Number of observations refers to the number of times the same type of breach committed by the same staff member was observed during the corresponding rotation. This allowed us to determine if the breach of confidentiality was an isolated or repeated incident, which in turn, had an effect on the degree of severity of the breach.

### Type of breach observed

Once all the templates were collected, the recorded breaches of confidentiality were classified into three categories according to their description as follows:

1. Confidentiality breaches related to the custody of clinical histories and records (admission forms, clinical and nursing report sheets, laboratory tests and other complementary examinations, and any other type of record containing patient data), as well as computer access to such records.
2. Confidentiality breaches related to the consultation and/or disclosure of clinical and/or personal data to medical personnel not involved in the patient’s clinical care, as well as people external to the hospital.
3. Situations in which the improper disclosure of the patient’s clinical data resulted from inadequate infrastructure, equipment, or poor organization of the hospital.

### Breach severity

In addition, we ranked the severity of the breaches described above from low to high severity as follows:

1. Minor confidentiality breaches are defined as those in which sensitive patient data is not properly safeguarded or handled (excluding the following categories), but which do not result in observable consequences. This includes the custody of clinical histories and records or breaches due to inadequate hospital infrastructure.
2. Minor confidentiality breaches committed repeatedly: more than once.
3. Severe confidentiality breaches are defined as the disclosure of sensitive data, as well as incidents that result in some kind of observable consequence. These breaches correspond to situations where clinical patient data are disclosed to third parties or to medical personnel not involved in the patient’s care, as well as those that are committed intentionally, or related to the patient’s sexual life, mental or other stigmatizing illnesses, and racial or ethnic background. Such breaches are considered to be particularly severe as these data are of a highly private nature.
4. Serious confidentiality breaches that occur repeatedly: more than once.

### Area where the breach was observed

In order to reduce the number of areas where the observations were recorded, we grouped the areas into categories based on their similarity as follows:

1. Meeting areas (offices, classrooms, etc.) and specific areas where healthcare is provided (exam rooms, treatment rooms, operating rooms, etc.).
2. Nursing stations on hospital wards.
3. Patient rooms, which are usually occupied by two patients and their respective companions.
4. Other public areas: corridors, elevators, hospital entrances, stairs, and locker rooms.

### Personnel involved in the breaches

The observers were required to record the staff member who committed the breach of confidentiality. Once all the data were collected, it was found that two or more staff were often responsible for the confidentiality breach. The personnel were classified as follows:

1. Physicians
2. Residents
3. Nursing staff
4. Nursing assistants
5. Orderlies
6. Administrative personnel
7. Students

### Frequency of observed breaches

Given that the observers were assigned different rotation periods during the academic year, the total hours of observation varied across medical departments (Table 1). Thus, a new quantitative variable broken down by medical department was used: the Frequency Index (FI). The FI indicates the number of confidentiality breaches recorded per hour of observation. To calculate the FI, the number of breaches committed in each department was averaged against the total hours of observation.

**Table 1:** List of observation periods in each medical department by academic years

Medical Departments	Total	
Internal Medicine and Emergency Department	D	1951
	H	9729
Gynecology and Obstetrics	D	1479
	H	6470
Pediatrics	D	1593
	H	7336
General and Digestive Surgery	D	1276
	H	6204
Maxillofacial Surgery and Plastic Surgery	D	563
	H	2551
Other Specialties	D	271
	H	858
Unknown	D	5
	H	9
Total	D	7138
	H	33157

D days, H hours

Statistical analysis of the data was performed using PASW Statistics 18 software (IBM SPSS®) for Windows. In addition to the descriptive analysis, proportions for the qualitative variables were compared between groups using chi-square tests (χ2) for contingency tables. For the FI quantitative variable, the comparison of means in the different medical departments was performed using the Kruskal-Wallis and Mann-Whitney U tests (post-hoc). Values above the 95 % confidence level (*p* < 0.05) were considered statistically significant.

## Results

Observations were conducted over a total of 7138 days and 33,157 h in the medical departments of the hospital during the study period. A total of 635 checklists with the observations recorded during the rotation periods were collected. Five of the confidentiality breaches reported by the observers were excluded from the study because some of the situations involved incidents not directly related to confidentiality. Specifically, these were cases where informed consent protocols were not properly followed or situations in which patient privacy was not violated because their clinical or personal data were discussed in the context of a clinical session to decide the most appropriate therapeutic approach to be taken. Finally, 630 questionnaires with valid observations were collected, of which 520 (82.5 %) referred to situations where patient confidentiality had been breached.

As regards distribution across medical departments, the largest number of checklists (25.2 %) and observed incidents (27.1 %) were collected in the Department of Internal Medicine and the Emergency Department. Pediatrics followed close behind with 24.3 % of all checklists and 21.2 % of recorded breaches. The lowest number of questionnaires and observed breaches corresponded to the “Unknown” category, with 0.8 % and 1 %, respectively.

### General characteristics of the observed breaches

The general characteristics of all the recorded confidentiality breaches, including their type and severity, where they were observed, and the personnel involved, are shown in Table 2.

**Table 2:** General characteristics of observed confidentiality breaches

	Number	Percent
*Type of breach* (*n* = 520)		
Custody of Clinical histories and records	179	34.4
Consultation/disclosure of clinical/personal data	284	54.6
Infrastructure breaches	57	11.0
*Breach severity* (*n* = 520)		
Minor	153	29.4
Minor breaches committed Repeatedly	75	14.4
Severe	243	46.7
Severe breaches committed Repeatedly	49	9.5
*Area where the breach was observed* (*n* = 520)		
Meeting and Specific areas	158	30.4
Nursing Stations	125	24.0
Patient Rooms	40	7.7
Other public areas	197	37.9
*Personnel involved in breaches* (*n* = 650)		
Physicians	334	51.4
Residents	122	18.8
Nursing Staff	130	20.0
Nursing Assistants	31	4.8
Orderlies	19	2.8
Administrative Personnel	7	1.1
Students	7	1.1

As regards the typology of the observed breaches, the most frequent were related to the consultation and/or disclosure of clinical and/or personal data to medical personnel not involved in the patient’s clinical care, as well as people external to the hospital. This type of breach accounted for 54.6 % of all recorded incidents.

As regards their severity, severe breaches were the most frequent, accounting for 46.7 % of all incidents.

Most of the reported incidents were observed in public areas (37.9 %), such as corridors, elevators, the cafeteria, stairs, and locker rooms.

With regard to the personnel involved in the confidentiality breach, 650 staff were responsible for 520 of the observed breaches. This is due to the fact that many of the incidents involved more than one person. Most of those responsible for the observed breaches were physicians, specifically 51.4 %.

### Frequency Index of breaches

**Fig. 1:**
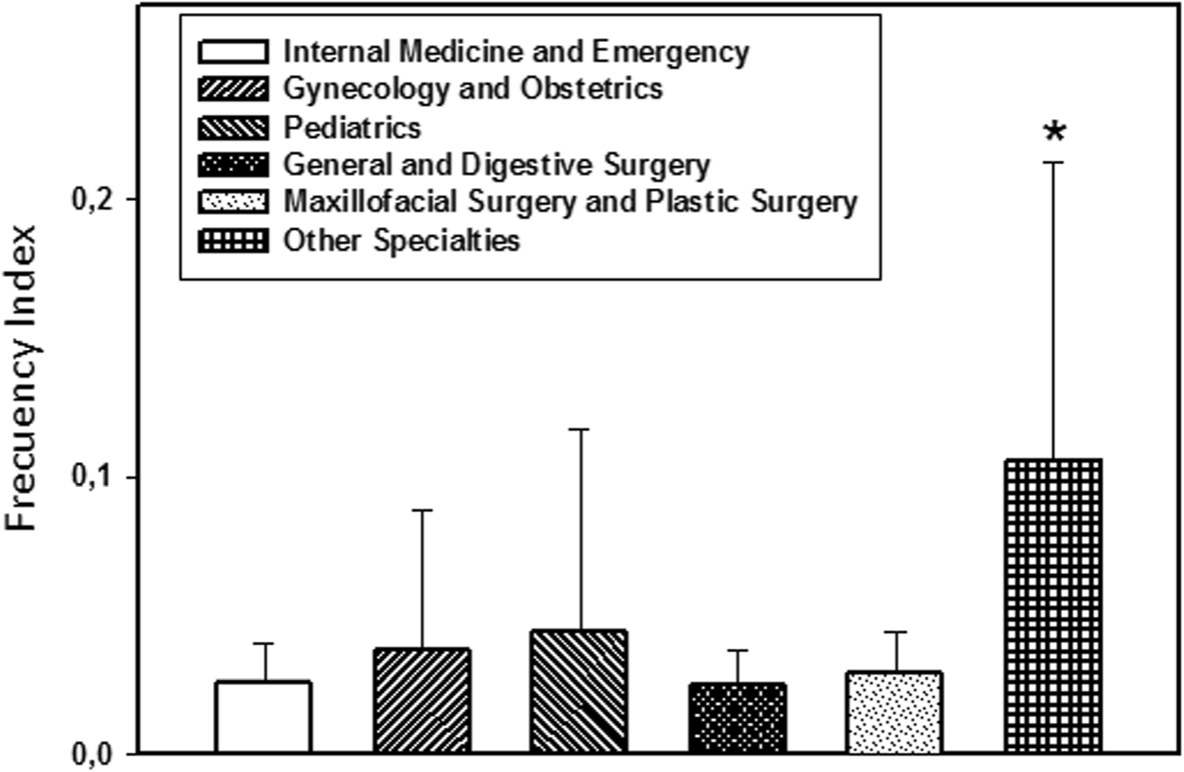
Frequency Index of confidentiality breaches observed in the medical departments (mean values; *: *p* < 0,001)

When calculating the FI for each medical department, the “Unknown” category was not taken into account as the small number of recorded observations did not allow us to determine the actual number of hours of observation, thus precluding the calculation of this index. As shown in Fig. 1, the calculations revealed that “Other medical and surgical specialties” had the highest median frequency of confidentiality breaches, with 0.083 breaches per hour of observation, while the lowest median IF corresponded to Internal and Emergency Medicine, with 0.023 confidentiality breaches per hour.

### Comparison by characteristics of the breaches

The “Unknown” category was excluded from the statistical analysis, in part due to the reasons mentioned above, but also because of the low incidence of confidentiality breaches recorded in these services (5). Therefore the calculations were performed on 625 rather than the 630 initial observations, and a total of 515 observed breaches were considered instead of 520.

No significant differences (*p* = 0.194) were found between observing a breach or not and the gender of the person making the observation.

The results for the association between medical departments and the personnel involved in the observed confidentiality breaches were statistically significant (*p* = 0.001). Across departments, physicians committed breaches of confidentiality most frequently, especially in Internal Medicine and the Emergency Department (54.8 %). Breaches were committed less frequently by the other groups; specifically, 24.8 % were committed by Internal Medicine and Emergency Department residents, and 30 % by Gynecology and Obstetrics nursing staff.

A statistically significant trend (*p* = 0.059) was found for the association between type of breach and the medical departments in which they were observed. In all cases, the most frequently observed breaches were those related to the consultation and/or disclosure of clinical and/or personal data to non-medical staff or third parties.

A statistically significant association was found for type of breach and the area of the hospital where it was observed (*p* < 0.001). As shown in Table 3, the most frequent breaches related to the disclosure to and/or consultation of clinical and/or data with non-medical staff and third parties were predominantly observed in meeting areas and specific work areas (75.8 %), patient rooms (90 %), and public areas (53.9 %). The most frequent breaches recorded at nursing stations were those related to the custody of clinical histories and documents (80 %).

**Table 3:** Relationship between type of confidentiality breach, area, and the personnel involved

*Type of breach* (*n* = 515) n (%)				
	CH^1^ (*n* = 175)	CP Dat^2^ (*n* = 283)	Infraest^3^ (*n* = 57)	p*
*Breach Area* n (%)				
Meet-Specif A^a^ (*n* = 157)	31 (19.7)	119 (75.8)	7 (4.5)	<0.001
Nurs. St^b^ (*n* = 125)	100 (80.0)	24 (19.2)	1 (0.8)	
Pat. Room^c^ (*n* = 40)	4 (10.0)	36 (90.0)	0 (0)	
Publ. A^d^ (*n* = 193)	40 (20.7)	104 (53.9)	49 (25.4)	
*Personnel involved in breaches* (*n* = 650) n (%)				
Physician (*n* = 334)	105 (31.4)	181 (54.2)	48 (14.4)	0.005
Resident (*n* = 122)	47 (38.5)	59 (48.4)	16 (13.1)	0.221
Nursing Staff (*n* = 130)	53 (40.8)	73 (56.2)	4 (3.0)	0.002
Nursing As (*n* = 31)	11 (35.5)	18 (58.0)	2 (6.5)	0.696
Orderly (*n* = 19)	13 (68.4)	6 (31.6)	0 (0)	0.004
Administrative P (*n* = 7)	2 (28.6)	5 (71.4)	0 (0)	0.553
Student (*n* = 7)	0 (0)	7 (100)	0 (0)	0.056

^1^Custody of clinical histories and records. ^2^Consultation/disclosure of clinical/personal data. ^3^Infrastructure breaches

^a^Meeting and specific areas. ^b^Nursing stations. ^c^Patient rooms. ^d^Other public areas

* Significance level. Contingency table Pearson’s chi-square test

Similarly, a statistically significant association was found between certain categories of personnel involved in the observed breach and type of breach (Table 3). Specifically, the association was significant for physicians (*p* = 0.005) and nursing staff (*p* = 0.002), with both groups being involved most frequently in the disclosure and/or consultation of clinical and personal data (54.2 % and 56.2 %, respectively). A statistically significant association was also found between orderlies (*p* = 0.004) and the custody of clinical records and histories (68.4 %).

**Fig. 2:**
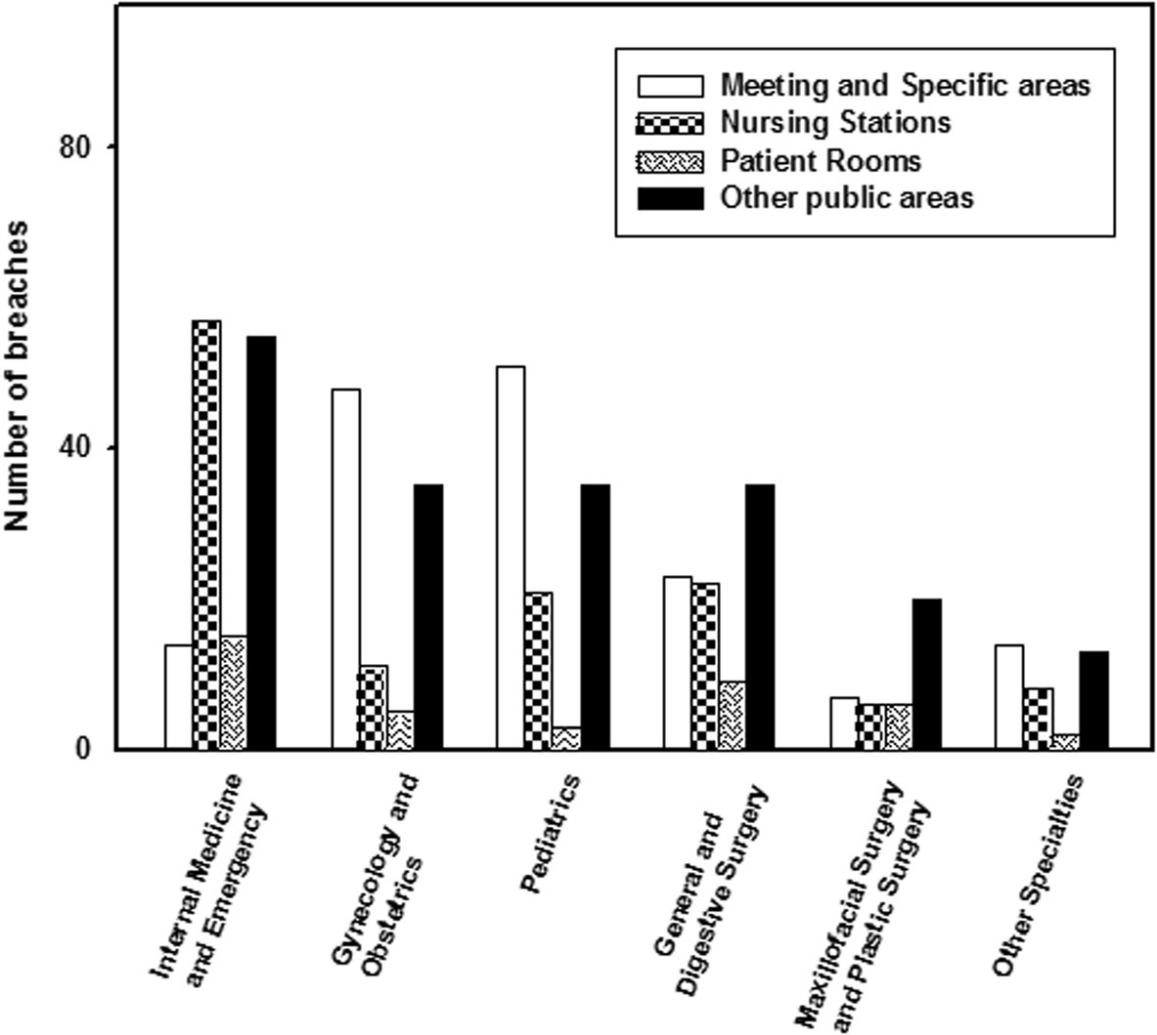
Relationship between area where confidentiality breaches were observed and medical departments

The association between areas of the hospital where breaches of confidentiality were observed and the medical department to which the person involved belonged was statistically significant (*p* < 0.001). As shown in Fig. 2, breaches of confidentiality were more frequent at the Internal Medicine and Emergency Department nursing stations (40.4 %), and in the meeting and work areas of Gynecology and Obstetrics (48.5 %) and Pediatrics (46.4 %). Breaches were observed more frequently in public areas corresponding to General and Digestive Surgery (39.3 %) and Maxillofacial and Plastic Surgery (51.3 %), and in meeting and specific work areas of other medical and surgical specialties (37.8 %).

**Fig. 3:**
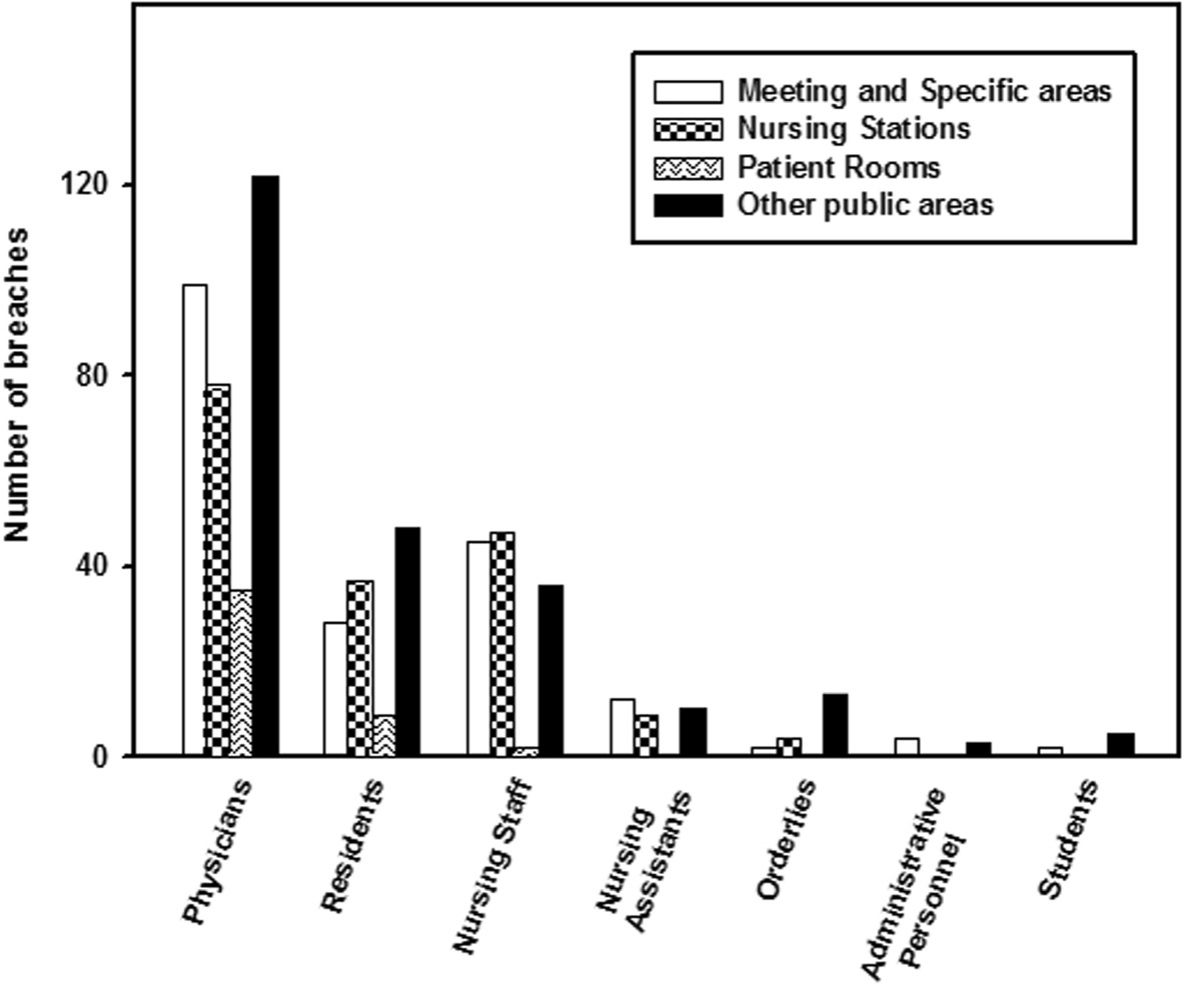
Relationship between area where confidentiality breaches were observed and personnel involved

Regarding the personnel involved in the breaches (Fig. 3), a statistically significant association was observed between physicians (*p* = 0.022) and orderlies (*p* = 0.026), both of whom committed the majority of breaches in public areas of the hospital (36.5 % and 68.4 %, respectively). A significant relationship (*p* < 0.001) was also found for nursing staff, with breaches primarily observed at nursing stations (36.2 %).

As shown in Table 4, there was a statistically significant relationship between the severity of the observed breaches and the medical department to which the person responsible for the confidentiality breach belonged (*p* < 0.001). The most frequent breaches were of a severe nature in all of the medical departments, particularly in other medical and surgical specialties (64.9 %) and Gynecology and Obstetrics (59.6 %).

**Table 4:** Relationship between breach severity, medical departments, area, and personnel involved

*Breach severity* (*n* = 515) n (%)					
	Minor (*n* = 149)	Min R^1^ (*n* = 75)	Severe (*n* = 242)	Sev R^2^ (*n* = 49)	p*
*Medical Departments* n (%)					
IM-E^a^ (*n* = 141)	36 (25.5)	41 (29.1)	46 (32.6)	18 (12.8)	<0.001
G-O^b^ (*n* = 99)	36 (36.4)	3 (3.0)	59 (59.6)	1 (1.0)	
PD^c^ (*n* = 110)	33 (30.0)	11 (10.0)	53 (48.2)	13 (11.8)	
G-D S^d^ (*n* = 89)	27 (30.3)	13 (14.6)	42 (47.2)	7 (7.9)	
M-P S^e^ (*n* = 39)	9 (23.1)	5 (12.8)	18 (46.2)	7 (17.9)	
O. S^f^ (*n* = 37)	8 (21.6)	2 (5.4)	24 (64.9)	3 (8.1)	
*Breach Area* n (%)					
Meet-Specif A^g^ (*n* = 157)	26 (16.6)	8 (5.0)	107 (68.2)	16 (10.2)	<0.001
Nurs. St^h^ (*n* = 125)	58 (46.4)	42 (33.6)	20 (16.0)	5 (4.0)	
Pat. Room^i^ (*n* = 40)	3 (7.5)	0 (0)	21 (52.5)	16 (40.0)	
Publ. A^j^ (*n* = 193)	62 (32.1)	25 (13.0)	94 (48.7)	12 (6.2)	
*Personnel involved in breaches* (*n* = 650) n (%)					
Physician (*n* = 334)	87 (26.0)	63 (18.9)	145 (43.4)	39 (11.7)	<0.001
Resident (*n* = 122)	32 (26.2)	29 (23.8)	47 (38.5)	14 (11.5)	0.006
Nursing Staff (*n* = 130)	33 (25.4)	23 (17.7)	60 (46.2)	14 (10.7)	0.543
Nursing As (*n* = 31)	9 (29.0)	2 (6.5)	15 (48.4)	5 (16.1)	0.389
Orderly (*n* = 19)	10 (52.6)	3 (15.8)	5 (26.3)	1 (5.3)	0.109
Administrative P (*n* = 7)	1 (14.3)	0 (0)	5 (71.4)	1 (14.3)	0.452
Student (*n* = 7)	0 (0)	0 (0)	6 (85.7)	1 (14.3)	0.138

^1^Minor breaches committed repeatedly. ^2^Severe breaches committed repeatedly

^a^Internal Medicine and Emergency Department. ^b^Gynecology and Obstetrics. ^c^Pediatrics. ^d^General and Digestive Surgery. ^e^Maxillofacial Surgery and Plastic Surgery. ^f^Other medical and surgical specialties. ^g^Meeting and specific areas. ^h^Nursing stations. ^i^Patient rooms. ^j^Other public areas

* Significance level. Contingency table Pearson’s chi-square test

Moreover, a statistically significant association was found between breach severity and the area of the hospital where the breach was observed (*p* < 0.001, see Table 4). Severe breaches were observed more frequently in meeting and specific work areas (68.2 %), while minor breaches were more frequent at nursing stations (46.4 %).

A significant association was observed within certain groups of personnel involved in the breach (Table 4), namely physicians (*p* < 0.001) and residents (*p* = 0.006), both of which committed severe breaches more frequently (43.4 % and 38.5 %, respectively).

### Comparison of FI between medical departments

A statistically significant association (*p* < 0.001) was found between the FI of other medical and surgical specialties and the remaining medical departments, with the former showing the highest frequency (Fig. 1).

## Discussion

The main objective of this study is to highlight the importance of patient confidentiality as a legal and ethical duty of health professionals in charge of patient care. To achieve this objective, and through a field study using many hours of direct observation (a total of 33,157 h), we have tried to reveal situations in which these professionals violate a duty inherent in their relationship with patients.

### How often is patients’ confidentiality breached?

To date, very few studies have directly recorded incidents related to confidentiality breaches during clinical practice in healthcare facilities, nor the frequency with which they occur. This last aspect, which we believe to be of great interest, was dealt with in a similar study by Mlinek and Pierce [11], who reported situations where patients’ confidentiality and privacy was breached in the emergency department of a university hospital with about 22,000 medical patient visits a year. Confidentiality breaches occurred for 26 out of 32 patients in the triage/waiting area over a 6 h observation period, whereas between 3 and 24 breaches occurred per hour in patient care areas during 18 h of observation.

Our study was conducted in a university tertiary hospital, but unlike the previous study, the observations were made in virtually all areas of the hospital; specifically 37 different CMUs. The observers recorded confidentiality breaches in all the departments, with a global FI of 0.016 breaches per hour (i.e., one confidentiality breach every 62.5 h). The median FI of confidentiality breaches (Fig. 1) was higher in the category of “other medical and surgical specialties”, where 1 breach for every 12.05 h of observation was recorded. This is probably due to the fact that although fewer total hours of observation were conducted, this category includes a larger number of CMUs. In 2012, the Emergency Department of the hospital involved in our study conducted 124,847 medical patient visits.^3^ Considering that our estimate was made jointly (Internal Medicine and the Emergency Department), the median of breaches was 1 per every 43.48 h of observation. Therefore, Internal Medicine and the Emergency Department, as well as General and Digestive Surgery were the departments with the lowest FI.

As can be seen, the average number of breaches we recorded was much lower than that reported by Mlinek and Pierce [11] (even considering our joint category). There are many additional reasons why both studies are not comparable. For example, Mlinek and Pierce [11] recorded a wide range of incidents that included comments and information obtained on patients through auditory and visual observation. Moreover, the observers in their study were specifically located in certain areas of the hospital chosen by the researchers themselves which are conducive to certain types of confidentiality breaches considered to be the most frequent. In contrast, our observers did not choose a particular area to “seek out” incidents either in the exams rooms or patient care areas of the Emergency Department. Another factor regarding the lower FI we report is that our observers received specific training using a checklist of the most common breaches, although this may have conditioned them to focus primarily on the breaches established by the researchers a priori.

### Characteristics of the confidentiality breaches in our hospital

The checklists completed by the observers included a record of the hours and days spent observing each medical department, as well as other information such as a description of the observed breach of confidentiality, the area of the hospital where it occurred, and the type of staff; factors that were taken into account when analyzing the recorded incidents.

Our study reveals that most confidentiality breaches (or incidents regarding a disclosure of confidential information) occurred primarily in public areas such as corridors, elevators, and stairs (37.9 %). Due to the presence of people external to the hospital in these areas, confidential information should be treated with utmost care. Indeed, one of the first fieldworks on the breach of confidentiality [10] already pointed in that direction. In their study, Ubel and Cols [10] made observations in 259 elevator rides in different hospitals, reporting inappropriate comments that breached patient confidentiality in 14 % of all rides. In our study, public areas were followed closely behind by work areas (30.4 %), medical consultations, treatment rooms, and operating rooms. This widespread phenomenon varied from one department to another and also depended on the type of breach.

Regarding the categories of confidentiality breaches we established, a large number were related to the custody of clinical records (Type 1). Specifically, there were situations in which folders containing medical records were left open on the counters of nursing stations where anybody walking by could see them, or left unguarded on carts in the middle of corridors and other public areas, and were even lost in such unlikely places as locker rooms, classrooms, or patients’ rooms. As for electronic clinical records, there was a number of cases where computers were left unguarded, thus allowing anyone to access them. The improper destruction of records with patient data such as throwing out the trash in public wastepaper baskets without destroying bracelets, identifying stickers, or patient lists occurred to a lesser degree.

The disclosure of clinical or personal data to non-medical staff or third parties (Type 2) was the most frequent type of breach (54.6 %), with situations in which the clinical and even personal data of identifiable patients or patients who had just left the physician’s office were discussed either in front of another patient, by phone, or with other colleagues not involved in the clinical assistance. Conversations in which specific data was revealed about patients were also frequent in public areas, especially corridors, stairs, and elevators. Another type of observed behavior was providing care in consultations or treatment rooms with open doors or curtains, conducting medical examinations of patients in their rooms on the ward in the presence of relatives of another patient who was in the room, and the retrieval of electronic data by an acquaintance not involved in the patient’s care without the patient’s knowledge or consent.

As for situations where confidentiality was breached due to inadequate infrastructure or poor organization (Type 3), the majority occurred when informing patients’ families in hospital wards, operating rooms, or unsuitable areas such as corridors and waiting rooms due to the lack of space. The observers also reported other situations in which practitioners decided to place several patients in the same room in order to conduct certain examinations due to the shortage of material.

In relation to the degree of severity, severe breaches were the most frequent (46.7 %). This is due to the fact that most incidents were related to the disclosure of clinical or personal data (Type 2), and were considered particularly severe with regard to protecting patient privacy. Breaches which led to some kind of observable consequence were also considered severe; for example, when conversations inside an exam room were overheard because the door was left open, and obviously when there was some intentionality in the action. These last cases, in which personnel breached the patient’s confidentiality in an intentional manner—by accessing electronic records to consult the clinical data of acquaintances who were not their patients and without the patient’s consent; or the case of the physician that disclosed information about a psychiatric patient to a representative of a pharmaceutical company at the entrance to an exam room−were fortunately rare. In most cases, we assume that the reasons for such breaches of confidentiality arise from a lack of knowledge about the legal and ethical repercussions of such actions, as well as carelessness in handling information. Our opinion is in line with studies such as that of Elger [12] who conducted surveys with groups of physicians. They found that although health professionals are often aware of the importance of confidentiality, a significant percentage does not how to avoid breaches of confidentiality in their daily practice.

We found that breaches defined as severe (68.2 %) (Table 4), and hence those that involve the disclosure of patients’ clinical and personal data (Type 2), were more frequent, particularly in meeting or work areas (75.8 %). This is not surprising as most patient care is provided in exam rooms, treatment rooms, and operating rooms where a large amount of data is handled. In contrast, incidents related to the custody of clinical histories (Type 1) were more frequent at nursing stations (80 %) as were minor breaches (46.4 %). This may be explained by the fact that most clinical records, either in paper or electronic format, are handled in these areas of the hospital. Specifically in the case of Internal Medicine and the Emergency Department, these incidents were more frequent at nursing stations (40.4 %) (Fig. 2). This is because the majority of breaches (43.3 %) involved the disclosure of data (Type 2), while a slightly lower percentage (39.7 %) was related to the custody of clinical records (Type 1). This is likely due to the fact that information regarding the patient’s clinical course, is often recorded at nursing stations, where unguarded folders containing clinical records may be left open on counters or displayed in computers without a password, thus permitting access to anyone passing by.

In relation to factors intrinsic to emergency departments, another study by Olsen and Sabin [13] reported that 36 % of patients and family members overheard conversations and that 1.6 % heard inappropriate comments, although they did not find significant differences between patients placed in walled vs. curtained rooms. In a subsequent study, Olsen and Cols [14] reported that after elimination of rooms separated only by curtains, the percentage of patients who overheard conversations between medical staff dropped to 14 %.

In Gynecology and Obstetrics (48.5 %), Pediatrics (46.4 %), and other medical and surgical specialties (37.8 %), a larger number of confidentiality breaches were observed in meeting and work areas (Fig. 2). This is consistent with the fact that the most common breaches in these areas were the disclosure of clinical or personal data to personnel not involved in the patient’s care or third parties (Type 2) as most medical care and personal contact with patients occurs in exam rooms, treatment rooms, and operating rooms. Physicians have often been reported to converse with colleagues about an identifiable patient in front of another patient in exam rooms or on the phone. In the surgical departments of our hospital (Fig. 2), such as General and Digestive Surgery (39.3 %) and Maxillofacial and Plastic Surgery (51.3 %), breaches of confidentiality were primarily observed in the public areas of the hospital. This may be due in part to the fact that, as our observers noted, it is common practice to inform family members in areas such as corridors and waiting rooms following surgery.

Another factor analyzed in our study were those responsible for breaches of confidentiality. Like Ubel and Cols [10] and Mlinek and Pierce [11], we found that such incidents were committed by all healthcare personnel, including, in our case, medical students. Hendelman and Byszewski [15] also demonstrated that medical students were involved in 19−51 % of all reported incidents.

In our study, physicians were observed to be responsible for the largest number of breaches (51.4 %), although we believe that this might be due to some bias as the observers were medical students who were doing their clinical internships primarily under the direction of physicians and to a lesser degree with medical residents. This is an important point because although medical care is currently provided by teams, and all members of the team have the obligation to maintain confidentiality, it is physicians who are primarily responsible for ensuring that this duty is met, not only with respect to patients’ clinical data, but also other types of information inherent to the doctor-patient relationship.

As regards the characteristics of the breaches (Table 3) in general, and especially in the case of physicians (54.2 %) and nurses (56.2 %), the most frequent had to do with the disclosure of clinical or personal data to non-medical staff or third parties (Type 2), and were therefore of a severe nature. In contrast, orderlies were responsible for most of the minor breaches (52.6 %) (Table 4) related to the custody of clinical histories (68.4 %) (Type 1, see Table 3). Regarding the personnel involved in breaches and breach severity, the collection of data was performed anonymously and the identity of the observed subjects was unknown, therefore we could only determine the number of repeated minor and severe breaches and the type of personnel involved in them, but not specifically how many different subjects were really responsible of the breaches. The main objective of our study is to examine real situations collecting general and sociodemographic data (medical departments, area, type of personnel involved…) in order to propose necessary measures to prevent such incidents, but devoid of any punitive intention.

As to the area where the breaches occurred (Fig. 3), breaches committed by nursing staff were observed primarily at nursing stations (36.2 %). This is not surprising as this is the area where they carry out much of their work. On the other hand, auxiliary (38.7 %) and administrative staff (57.1 %) were observed to commit most breaches in meeting and work areas as they perform their tasks primarily in offices. As regards the rest of the hospital staff, especially physicians (36.5 %) and orderlies (68.4 %), breaches were committed most frequently in public areas. In the case of physicians, this could be explained by careless behavior, and because they are primarily responsible for informing patients and their families, which, as mentioned above, is often done in public areas such as corridors and waiting rooms. With regard to orderlies, breaches are mainly committed in public areas as one of their principle tasks is to transfer clinical records. As the observers repeatedly noted, “medical records were found lying about unguarded in hospital corridors”.

### Limitations of the study

Among the limitations of our study, we should first note that the observers selected for the fieldwork were medical students. This could have had an effect on the recorded observations since their knowledge and expertise on the subject was, to some extent, limited. However, we attempted to overcome this limitation by providing personalized training to each of the observers.

In addition, although the observers signed a confidentiality agreement to avoid suspicion of being observed and the subsequent bias of changing their behavior, we cannot completely rule out the possibility of a Hawthorne effect as a confounding factor.

Moreover, the type of breaches recorded by the observers were subjectively classified a posteriori into specific categories based on the content of the comments. In cases deemed to be unclear, consensus was reached among the researchers regarding the category in which to include the breach.

On the other hand, the study was carried out in a Spanish university tertiary hospital, and though we do believe that the problem is very similar in other hospitals, it cannot be directly generalized.

Finally, it should be noted that other medical and surgical specialties was not a homogeneous category as it was comprised of different CMUs that were grouped together for the purpose of statistical comparison.

## Conclusions

The breach of patient confidentiality remains one of the major problems encountered in daily clinical practice. Following many hours of observation in a tertiary hospital, we found an estimated Frequency Index of one breach per 62.5 h. Confidentiality breaches are important due to the consequences they have for the doctor-patient relationship, and because the lack of security of private patient information may have social implications that could eventually translate into a loss of confidence in the healthcare system.

In addition to aspects related to hospital organization or infrastructure, we have shown that all healthcare personnel are involved in confidentiality breaches, especially physicians (the most frequent group). While most are committed unintentionally, a non-negligible number are severe, repeated breaches (9.5 %), thus suggesting certain carelessness, perhaps through ignorance about certain behaviors that can jeopardize patient confidentiality. Our findings indicate that it is advisable to improve medical education about the importance of confidentiality at both the undergraduate level and through awareness campaigns among medical professionals that stress the need for greater care and attention in the management and handling of clinical information.

## Additional file

Additional file 1: STROBE document. (DOCX 34 kb)

## Footnotes

1 These data were obtained from the 2012 Annual Report.

2 Strobe document with items that should be included in reports of cross-sectional studies

3 These data were obtained from the 2012 Annual Report. Clinical Management Unit of the Adult Emergency Department.
